# Gait Pathology in Subjects with Patellofemoral Instability: A Systematic Review

**DOI:** 10.3390/ijerph191710491

**Published:** 2022-08-23

**Authors:** Andreas Habersack, Tanja Kraus, Annika Kruse, Katharina Regvar, Michael Maier, Martin Svehlik

**Affiliations:** 1 Department of Orthopaedics and Trauma, Medical University of Graz, Auenbruggerplatz 5, 8036 Graz, Austria; 2 Institute of Human Movement Science, Sport and Health, University of Graz, Mozartgasse 14/I, 8010 Graz, Austria; 3 AUVA Trauma Centre Styria, Göstingerstrasse 24, 8020 Graz, Austria

**Keywords:** patellofemoral instability, 3D gait analysis, gait alterations

## Abstract

Identifying potential gait deviations in patellofemoral instability (PI) can help with the development of effective rehabilitation strategies. The purpose of this systematic review was to examine whether there are specific gait alterations in subjects with PI. The present review followed the PRISMA guidelines and was initially registered at PROSPERO (CRD42021236765). The literature search was carried out in the databases of PubMed, the Cochrane library, Web of Science, ClinicalTrials.gov, and Medline. The search strategy resulted in the identification of seven relevant publications. Subjects with PI show decreased walking speed, stride length, and cadence. Some studies reported changes not only in knee kinematics and kinetics but also in hip and ankle kinematics and kinetics. There is evidence that most subjects with PI walk with a quadriceps avoidance gait and show increased genu valgum posture, but there is still great variability in the coping responses within individuals with PI. The discrepancy among the study results might underpin the fact that PI is a multifactorial problem, and subjects cope with the different underlying morphological as well as functional deficits using a variety of gait strategies, which makes the interpretation and understanding of the gait of subjects with PI a clinically challenging task.

## 1. Introduction

Patellofemoral instability (PI) is known as an individual or combined deficiency of bony structures, ligaments, and neuromuscular factors that result in the patella becoming misaligned in the trochlear groove as the knee flexes and extends. During extension movement of the knee joint, the patella enhances the power transmission of the extensor muscles due to a longer lever arm [1,2]. However, a deficiency of the described structures results in a higher risk of dislocating the patella partially or completely from the trochlear groove [3].

Patellar instability primarily affects young, active females during physical activity [4]. The most common dislocation occurs in the knee flexion range up to 30° with an external rotation of the tibia and contraction of the quadriceps, which can be found in various sports, particularly in ball and “pivoting” sports [5].

There are different factors that increase the risk of an unstable patellofemoral joint, such as an insufficient medial patellofemoral ligament (MPFL), lateralized tibial tuberosity, patella alta, trochlear dysplasia, or genu valgum [3]. If the patella has already been dislocated, the presence of two or more factors (e.g., patella alta and trochlear dysplasia) can increase the risk of recurrent instability by more than 50% [6]. Between 30% and 80% of patients with a primary episode of PI have recurrent dislocations, with females at a higher prevalence rate than males [7,8].

Symptoms of PI include a feeling of instability and/or anterior pain at the knee, which can be functionally debilitating and limits the ability to conduct sports and exercise [9]. Approximately half of the patients with PI experience early patellofemoral osteoarthritis symptoms, including swelling, quadriceps weakness, and pain when navigating stairs [10,11].

If the patella is dislocated for the first time and there are low-risk factors for recurrence, PI can be conservatively treated by immobilization and accompanying physiotherapy [9,12]. However, if there are chondral injuries, increased risk factors (e.g., pivoting sports), or other factors such as trochlea dysplasia, lateralization of the tibia tuberosity, or MPFL lesion, surgical measures may be necessary [9]. For surgical planning, clinical examination and magnetic resonance imaging (MRI) are used to decide which surgical strategy should be pursued [13]. Due to the high risk of redislocation [7,8], 3D gait analysis, if available, can be considered as an additional examination method for surgical planning so as to gather even more detailed information about the functional limitations that may affect orthopedic decision-making (e.g., surgical planning of rotational osteotomies of the femur and tibia) [14,15,16].

However, it still remains unclear how and if gait pattern alterations in patients with PI increase or decrease the risk of patellar dislocation [17]. Due to the multifactorial interplay of all the above-mentioned factors and the varying morphological and functional presentation, the interpretation and understanding of gait patterns and compensatory strategies for subjects with PI is a clinically challenging task [18].

Several studies investigated the gait pattern in subjects with PI. These studies revealed that subjects with PI show, for instance, decreased gait velocity and cadence and greater internal knee abduction moment than healthy subjects [19,20,21,22,23,24,25,26,27,28]. However, some of the studies reported inconsistent/contradictory results, which may indicate the existence of different compensatory gait strategies within subjects with PI due to the multiple influencing factors [29].

The aim of this systematic review was to review the current knowledge and obtain a better insight into the possible gait alterations of individuals with PI. The aim is presented in more detail in Table 1 by means of the “population, intervention, comparison, outcome (PICO)” scheme [30]. A better understanding and interpretation of the gait alterations and compensatory strategies in subjects with PI is essential to develop effective treatments and a reduction in recurrence and attendant symptoms in adulthood.

## 2. Materials and Methods

### 2.1. Search Strategies

This systematic review was carried out according to the PRISMA guidelines [31]. The protocol for this systematic review was registered at PROSPERO (registration number: CRD42021236765) and is available in full on the NIHR HTA Programme website (www.crd.york.ac.uk/prospero accessed on 16 August 2022). A comprehensive literature search was conducted in the following electronic databases: PubMed, the Cochrane library, Web of Science, ClinicalTrials.gov, and Medline. The search was started in April 2021 and finalized in May 2021. In addition, unpublished data from the local hospital gait laboratory database and reference lists of retrieved relevant articles were also considered. No restriction on the publication date was made.

Appropriate search terms were identified prior to initiating the search. The search terms used were “(gait) AND (patella) AND (instability OR luxation OR subluxation OR dislocation)”.

### 2.2. Eligibility Criteria

All study types (randomized controlled trials, cross-sectional studies, etc.) were included if they described participants who were diagnosed with patellofemoral instability, patella luxation, or patella subluxation and examined by means of a 3D motion capture system measuring spatio-temporal parameters and lower body kinematic, and/or kinetic gait parameters. Exclusion criteria were studies that included subjects with comorbid conditions previously identified to cause PI or subjects who had already undergone patella-stabilizing surgery before the 3D gait analysis was conducted. Table 2 depicts the inclusion and exclusion criteria for this systematic review.

### 2.3. Selection of Studies and Data Synthesis

After the preliminary search, article titles were screened for duplicates and topic relevance. Two reviewers screened the abstracts of the remaining studies. Based on the abstracts, the investigators obtained copies of potential full-text articles and assessed the eligibility for the final inclusion. One reviewer independently extracted the data. Included variables were any measures that assessed spatio-temporal, kinematic, or kinetic gait parameters. Even if a study reported only one of the three mentioned gait parameters, the article was still included in this review. The included gait parameters were analyzed and compared with regard to the respective joints (hip, knee, ankle).

### 2.4. Quality Assessment

In order to assess the methodological quality of the included full-text articles and to avoid bias across the publications, the scientific papers were analyzed following the Strengthening the Reporting of Observational Studies in Epidemiology (STROBE) checklist for reporting cohort, case-control, and cross-sectional studies (combined) [32,33]. The checklist recommends all the important steps for successful scientific reporting and helps to establish whether the relevant information is presented clearly or not. A “1” in Table 3 illustrates that the criterion in the checklist was satisfied and presented clearly; a “0” demonstrates that this was not the case.

## 3. Results

### 3.1. Search Results

**Figure 1:**
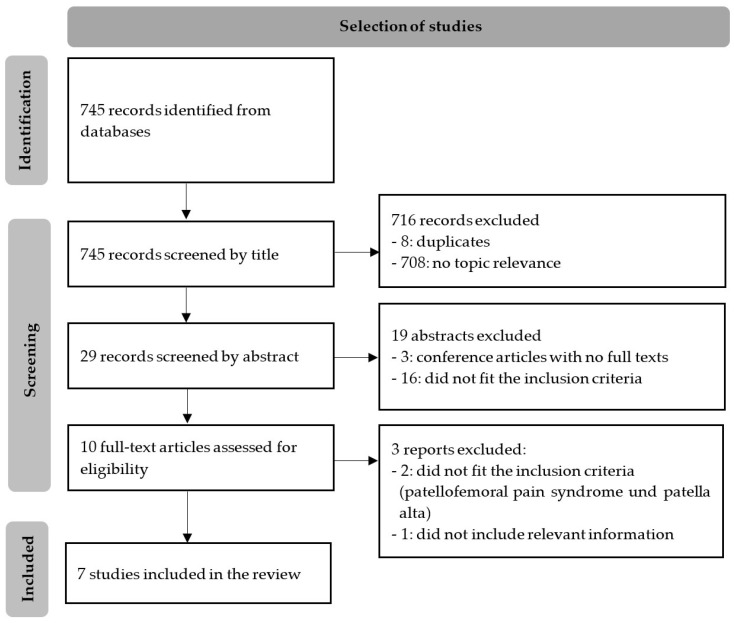
Adapted flowchart demonstrating the selection of articles through the review process [31].

The database search was completed in May 2021 and resulted in 745 records (83 PubMed, 19 the Cochrane library, 48 Web of Science, 593 Medline, 2 ClinicalTrials.gov). Following the identification of duplicates, all authors shared the screening of the titles and the subsequent abstracts, resulting in 10 articles being identified. The full-text screening was then performed by two authors, and seven articles were deemed to meet the inclusion criteria. These articles were subsequently used in the analysis. Systematic reviews were not found during the search process. The flowchart demonstrating the selection of articles is shown in Figure 1.

### 3.2. Methodological Quality

Almost all the studies [24,25,26,28,29,34] achieved a total score of 18 or higher out of a possible total of 22 points for the STROBE checklist. There is only one study by Sowiński et al. [27] where nine of the 22 recommended points of the STROBE checklists were missing. Two studies failed to report the specific demographic information of their participants [25,27]. Only two studies [28,34] reported how they derived their sample size. The results of the critical appraisal for each article are displayed in Table 3.

### 3.3. Study Characteristics

The included studies were conducted in different parts of the world, including Poland [27], the USA [25], Austria [26,34], the UK [29], and Switzerland [24,28].

The age of participants in the studies ranged from approximately 12 to 35 years.

Of the seven included studies, one had a pre-post comparison design [28], two were assessed retrospectively [26,34], and four had a cross-sectional study design [24,25,27,29]. Only two studies reported spatio-temporal parameters [24,27], whereas all of the studies reported kinematic gait parameters [24,25,26,27,28,29,34]. Six studies also reported kinetic gait variables [24,25,26,28,29,34]. A summary of the assessed gait variables is provided in Table 4.

### 3.4. Gait Parameters

#### 3.4.1. Spatio-Temporal Parameters

Patients with PI showed decreased walking speed [24,27], stride length [24], and step frequency [27]. The duration of their midstance phase was reduced, whereas the duration of the loading response was prolonged, resulting in an increased duration for the double support phase [24].

#### 3.4.2. Joint Characteristics

##### Hip

From a kinematic point of view, subjects with PI showed an external rotation of the hip in the transverse plane [26] and reduced hip flexion during the whole gait cycle [24]. There was a considerable kinetic difference observed in the two studies with respect to hip abduction moment. Schranz et al. [26] found increased hip abduction moment, which is in contrast to the findings of Lucas et al. [25], where subjects with PI demonstrated reduced peak abduction moment. Subjects with PI also showed decreased hip flexion moment during the stance phase, which is in accordance with the reduced hip flexion [24]. All the other studies [27,28,29] did not report any significant alteration in gait pattern with respect to the kinematic and kinetic course of the hip.

##### Knee

The kinematic variables of the knee described in the studies were diverse, and the findings were sometimes contradictory. For instance, Clark et al. [29] found that subjects with PI walked with a slightly flexed knee, avoiding extension, whereas Lucas et al. [25] reported no difference in knee kinematics compared to healthy controls. Camathias et al. [24] and Ammann et al. [28] observed decreased knee flexion during the entire gait cycle, while Sowiński et al. [27] found hyperextension of the knee during the stance phase. In accordance with these three studies [24,27,28], Schranz et al. [26] observed reduced peak values of the sagittal knee angle during the stance and swing phase. However, in a second study by Schranz et al. [34], increased as well as decreased knee flexion during the stance phase was found, which confirms the inconsistent results for knee movement in the sagittal plane in individuals with PI.

Schranz et al. [26] further reported increased internal rotation of the knee and a genu valgum posture. Lucas et al. [25] also reported decreased knee adduction angles, which promotes the occurrence of valgus posture in subjects with PI.

Subjects with PI showed increased knee abduction moment [26], reduced sagittal knee moment during the initial midstance [24], and decreased knee extension moment [28]. In contrast, Lucas et al. [25] observed no difference in knee extensor moment, while Schranz et al. [34] even found increased knee extensor moment during the stance phase in a subgroup of subjects with PI.

##### Ankle

Schranz et al. [26] observed internal rotation of the ankle and increased external tibia rotation. Only one study by Camathias et al. [24] reported both sagittal ankle kinematics and kinetics. In this study, subjects with PI showed increased plantar flexion and plantar flexion moment during initial contact and loading response [24]. All the other studies [25,27,28,29,34] did not report any significant alteration in gait pattern with respect to the kinematic and kinetic course of the ankle compared to healthy individuals.

## 4. Discussion

The aim of this systematic review was to review and compile the current knowledge on potential gait alterations in subjects with PI to obtain a better understanding of the impact of PI on gait function.

This is the first review to investigate gait pathology in subjects with PI. A marked discrepancy in the results of several studies was observed with respect to the differences in many kinematic and kinetic parameters. This might underpin the suggestion that PI is a multifactorial problem that depends on the architecture and structure of the patella and trochlea, limb alignment, soft tissue properties, and the interplay and coordination of the synergist and antagonist muscles, which influences the morphological as well as the functional appearance of gait in individuals with PI [18,34].

Although only two studies [24,27] reported spatio-temporal parameters, these findings were consistent, revealing a clear reduction in gait speed, stride length, and step frequency. Since these parameters are easy to collect and form the basis of every gait analysis, we recommend collecting and reporting these parameters in addition to the kinematic and kinetic parameters in future studies.

The kinetic and kinematic parameters of the knee seem to be an indicator that can be used to describe gait pathology in subjects with PI. Sowiński et al. [27] observed hyperextension of the knee during the stance phase. However, due to the small sample size (*n* = 6), this finding cannot be generalized and should be interpreted with caution. Nevertheless, due to the high number of samples in the studies of Camathias et al. [24], Amman et al. [28], and Clark et al. [29] (overall *n* = 100), it seems reasonable to assume that subjects with PI have something in common, i.e., the quadriceps avoidance gait. Camathias et al. [24] and Ammann et al. [28] observed that subjects with PI walked with a less flexed knee during the entire gait cycle. We suspect that the anxiety associated with instability, knee pain, and limited range of motion may be the reason why subjects with PI do not flex their knee as much as typically developing peers.

The decreased knee flexion during the stance phase was also investigated in several studies dealing with anterior cruciate ligament deficiencies or patellofemoral pain syndrome [19,22,35,36,37,38,39,40,41]. A quadriceps avoidance gait was found in 75% of all anterior cruciate ligament (ACL) knees [35]. In ACL deficiencies, this might be a protective mechanism. The quadriceps avoidance gait may protect the knee against the anterior translational displacement of the tibia that would result in joint instability or pivoting, as the quadriceps has a tendency to shift the tibia between a range of 0° and 45° knee flexion [24,42]. However, as the patella enters the trochlea groove in a 10–30° flexed knee position [12,43], the extended knee position might bring further instability in subjects with PI if soft tissue stabilators are missing (e.g., MPFL lesion). In addition, the lack of normal knee flexion and even the hyperextension patterns throughout the stance phase observed in subjects with PI [24,27,28,29] could have serious consequences for the knee joint. During the initial contact and loading response, the physiologic knee flexion acts like a damper absorbing the shock from the impact to reduce peak compression forces in the knee joint. In contrast, a limb that is instead hyperextended during these phases of the gait cycle directly transfers the body weight and the shock from the femur to the tibia, resulting in abnormally increased contact force in the tibiofemoral joint.

In accordance with the quadriceps avoidance gait, Lucas et al. [25] additionally reported that subjects with PI were significantly weaker in muscle strength during knee extension, which contributes to the suggestion of weaker quadriceps muscles and the quadriceps avoidance gait.

In addition, several studies observed an increased knee valgus angle. Due to the relative position of the hip, knee, and ankle, the increased knee valgus angle could potentially contribute to lateral tracking of the patella, which might increase the risk for recurrence of a patellar dislocation [25]. With the increased external tibial rotation found by Schranz et al. [34], this would result in a high lateral displacement force on the patella and a considerable risk of dislocation, which can frequently be observed in most sports types.

Interestingly, only a few studies reported significant gait pathology regarding hip and ankle kinematics and kinetics. Furthermore, there were no common and overlapping results across the included papers. Schranz et al. [26] reported increased hip abduction moment, which contradicts the findings of Lucas et al. [25], who found that subjects with PI showed reduced peak hip abduction moment. Subjects with PI also presented decreased hip flexion moment and reduced hip flexion during the stance phase [24]. Camathias et al. [24] observed that individuals with PI walked with decreased foot dorsal extension during initial contact and loading response. This mechanism could be a component of the quadriceps avoidance gait as the reduced ankle dorsal extension may increase the plantar-flexion/knee-extension coupled mechanism and thus reduce the force of the quadriceps during walking. It could also be suggested that the reduced ankle dorsiflexion and the decreased hip flexion during the stance phase may be a sign of knee stiffening to reduce the knee moment. This compensatory mechanism was shown to be beneficial in increasing knee stability in subjects with knee osteoarthritis [21]. Even if this strategy of avoidance helped to stabilize the patella during walking, it would not be successful in stabilizing the knee in high-velocity activities such as running or sports. However, it is debatable if this strategy is a compensatory mechanism or a simple adjustment and alignment of the interplaying hip, knee, and ankle joints.

As the results of this review reveal some conflicting findings, gait alterations should be further investigated in follow-up studies with a higher number of participants and datasets to reveal detailed information on gait pathology in individuals with PI. We hypothesized that the diverse gait patterns might also be due to the different causes of patellar instability, which should be reported with gait data, combined with risk factors and morphological classification. Furthermore, future studies should include specific demographic information (e.g., activities of daily living and sports and the subjective level of fear during walking), medical history, and a precise description of the morphological changes with regard to patellofemoral instability. We additionally recommend that future gait analyses of subjects with PI should also include electromyography (EMG) measurements to be able to capture the dynamic muscle activity, to support the finding of the quadriceps avoidance gait. In addition, we suggest conducting MRI measurements of the knee joints to explore the relationship between different gait pathologies and compensatory mechanisms. A better understanding of why this population walks with a specific pattern will make it easier for clinicians to appropriately treat each patient individually.

### Limitations

Although this systematic review was performed with the greatest care, there are certain limitations. Firstly, we reported only on the kinematic, kinetic, and spatio-temporal parameters. However, gait is influenced by a multitude of factors, such as the passive range of motion of the respective joints (e.g., knee flexion contracture), muscle strength, neuromuscular control, etc. It would be helpful to determine a holistic approach to walking ability by including additional parameters and correlating the influencing factors.

Since the effect of speed on gait variables is well known [44], and only two of the included studies reported on spatio-temporal parameters, the comparisons between the studies must be interpreted with caution. Gait speed affects the amplitude of spatio-temporal parameters, joint kinematics, joint kinetics, and ground reaction forces, decreasing in most values at slower speeds and increasing at faster speeds [44]. Only one study by Lucas et al. [25] controlled the gait speed (1.5 m/s), and in all the other studies, the participants walked at a self-selected speed. Therefore, the gait speed should be reported and considered in future studies, especially when interpreting and comparing the gait analyses of individuals with pathologies with control analyses [44].

Another limitation is that this review did not complete a quantitative meta-analysis due to the incomplete and wide range of gait parameters reported across the studies. In addition, due to the observational nature of most of the studies, the generalizability of their findings is limited, and they should be interpreted with caution. Even if the search strategy was implemented in a way to avoid publication bias (such as reference lists and personal files), due to the complexity and imprecise nature of searching and identifying, some publications may have been missed.

## 5. Conclusions

Subjects with PI seem to walk with decreased gait speed, stride length, and step frequency. Most individuals with PI show a quadriceps avoidance gait pattern, with a more outstretched knee combined with an increased genu valgum posture; however, there is high variability in the coping responses to PI.

Due to the varying functional presentations, the interpretation and understanding of gait alterations and the compensatory strategies of subjects with PI is still a clinically challenging task. The underlying causes and variability of the different gait patterns should be investigated in future studies.

## Figures and Tables

**Table 1:** Research question of this systematic review based on population, intervention, control, and outcome (PICO) [30].

Population	Intervention	Comparison	Outcome
Individuals with patellofemoral instability	No	Individuals with no history of patellofemoral instability	- Spatio-temporal parameters (step length, stride length, cadence, gait velocity, etc.) - Kinematic parameters (sagittal, transverse, and frontal plane angles of hip, knee, and ankle) - Kinetic parameters (ground reaction forces, joint moments of hip, knee, and ankle)

**Table 2:** Inclusion and exclusion criteria utilized to select articles.

Inclusion Criteria	Exclusion Criteria
patellofemoral instability kinetic gait parameters AND/OR kinematic gait parameters AND/OR spatio-temporal gait parameters	any other condition previously identified to cause patellofemoral instability (e.g., knee osteoarthritis or other functional limitations or disabilities) patella-stabilizing surgery before gait analysis had been conducted

**Table 3:** Results of the assessment of the methodological quality following the STROBE checklist for reporting cohort, case-control, and cross-sectional studies (combined). A “1” in the table illustrates that this criterion was satisfied and presented clearly; a “0” demonstrates that this was not the case. The introduction section includes “background and objectives”. The methods section includes “study design, setting, participants, variables, data sources, bias, study size, quantitative variables, and statistical methods”. The results section includes “participants, descriptive data, outcome data, main results, and other analysis”. The discussion section includes “key results, limitations, interpretation, and generalizability”. The other information section contains information about “funding”.

	Sowiński et al. [27]	Schranz et al. [26]	Lucas et al. [25]	Clark et al. [29]	Camathias et al. [24]	Schranz et al. [34]	Ammann et al. [28]
Title/abstract	1	1	1	1	1	1	1
Introduction	1/1	1/1	1/1	1/1	1/1	1/1	1/1
Methods	0/0/1/1/1/ 0/0/1/1	1/1/1/1/1/ 1/0/1/1	0/1/1/1/1/ 1/0/1/1	0/1/1/1/1/ 1/0/1/1	1/1/1/1/1/ 1/0/1/1	1/1/1/1/1/ 0/1/1/1	1/1/1/1/1/ 1/1/1/1
Results	0/0/1/1/0	1/1/1/1/0	0/1/1/1/0	1/1/1/1/1	1/1/1/1/1	1/1/1/1/1	1/1/1/1/1
Discussion	1/0/1/0	1/1/1/1	1/1/1/1	1/1/1/1	1/1/1/1	1/1/1/1	1/1/1/1
Other information	1	0	1	1	0	0	1
Total score	13/22	19/22	18/22	20/22	20/22	20/22	22/22

**Table 4:** Summary of the study characteristics of the articles included in this review. Abbreviations: TD = typically developing; ↓ = decreased; ↑ = increased; * = information from figures.

Authors	Participants	Parameters			Results (Compared to TD or Control)
		Spatio-Temporal	Kinematic	Kinetic	
Sowiński et al. [27]	6 subjects aged 17 to 29 years (4 males/2 females) with unilateral recurrent lateral patella luxation	x	x		- Gait speed ↓ - Step frequency ↓ - Knee extension ↑ (hyperextension)
Schranz et al. [26]	30 adolescents (2 males/28 females) with recurrent patellar instability (minimum three patella dislocations) aged 12 to 18 years		x	x	- Transverse hip angle: external rotation ↓* - Transverse knee angle: internal rotation ↑ * - Maximum sagittal knee angle during stance and swing phase: ↓ * - Frontal knee angle: valgus ↑ * - Transverse ankle angle: internal rotation ↑ * - Internal frontal hip abduction moment ↑ * - Internal frontal knee abduction moment ↑ *
Lucas et al. [25]	32 subjects (16 PI, 16 controls, 3 males/13 females in each group, mean age 21.1 years)		x	x	- Peak knee adduction angle ↓ - Valgus position ↑ - Internal peak hip abduction moment ↓
Clark et al. [29]	13 subjects with PFI (6 males/7 females, mean age 25.9 years) and 8 control subjects (5 males/3 females, mean age 24.8 years)		x	x	- Quadriceps avoidance gait (only slightly flexed knee during entire gait cycle (avoiding extension)) - Internal knee flexor moment ↑
Camathias et al. [24]	67 patients (88 knees) with recurrent patellar dislocation (25 males/42 females, mean age 14.8 years) and 27 healthy individuals as controls (54 knees) (13 males/14 females, mean age 14.9 years)	x	x	x	- Gait speed, stride length, duration of midstance phase ↓ - Loading response and time for double support ↑ - Hip flexion during entire gait cycle ↓ - Knee flexion during entire gait cycle ↓ - Ankle plantar flexion during initial contact and loading response ↑ - Ankle dorsal extension during initial contact andterminal midstance phase ↓ - Hip flexion moment from initial contact to terminal midstance phase ↓ - Sagittal internal knee moment during initial midstance phase ↓ - Ankle plantar-flexion moment during initial contact and loading response ↑
Schranz et al. [34]	42 patients (5 males/37 females, from 12 to 18 years) with recurrent patellar dislocation (at least 3 dislocations); 52 legs Divided into three groups based on their sagittal knee moment: - Patella unloading group (PUG) (19) - Patella overloading group (POG) (12) - Patella norm-loading group (PNG) (21)		x	x	All three groups: - External tibia rotation ↑ PUG: - Knee flexion and internal extension moment during stance phase ↓ - No positive sagittal internal knee extensor moment during weight acceptance phase POG: - Knee flexion and internal extension moment during stance phase ↑ PNG: - No adaption of gait pattern
Ammann et al. [28]	6 patients with bilateral patellar dislocation (6 females, mean age 14.6 years) and 14 patients with unilateral patellar dislocation (1 male/13 females, mean age 14.9 years). Control group of 27 subjects (14 males/13 females, mean age 14.8 years)		x	x	- Knee flexion during entire gait cycle ↓ * - Internal knee extensor moment during stance phase ↓ *
			Only knee	Only knee	

## Data Availability

Not applicable.

## References

[1] Melvin S.J., Mehta S. (2011). Patellar Fractures in Adults. J. Am. Acad. Orthop. Surg..

[2] Steinmetz S., Brügger A., Chauveau J., Chevalley F., Borens O., Thein E. (2020). Practical guidelines for the treatment of patellar fractures in adults. Swiss Med. Wkly..

[3] Post W.R., Fithian D.C. (2018). Patellofemoral Instability: A Consensus Statement from the AOSSM/PFF Patellofemoral Instability Workshop. Orthop. J. Sports Med..

[4] Hurley C.R.K., Rush M.J.K. (2015). Patellar instability in children and adolescents. Curr. Orthop. Pract..

[5] Höhne S., Gerlach K., Irlenbusch L., Schulz M., Kunze C., Finke R. (2017). Finke, Patellaluxation bei Kindern und Jugendlichen—136 Ereignisse bei 88 Patienten und Literaturübersicht. Z. Orthop. Unf..

[6] Parikh S.N., Lykissas M.G., Gkiatas I. (2018). Predicting Risk of Recurrent Patellar Dislocation. Curr. Rev. Musculoskelet. Med..

[7] Alaia M.J., Cohn R.M., Strauss E.J. (2013). Patellar instability. Bull. Hosp. Jt. Dis..

[8] Camp C.L., Heidenreich M., Dahm D.L., Stuart M.J., Levy B.A., Krych A.J. (2015). Individualizing the Tibial Tubercle-Trochlear Groove Distance: Patellar Instability Ratios That Predict Recurrent Instability. Am. J. Sports Med..

[9] Frosch S., Balcarek P., Walde T., Schüttrumpf J., Wachowski M., Ferleman K.-G., Stürmer K., Frosch K.-H. (2011). Die Therapie der Patellaluxation: Eine systematische Literaturanalyse. Z. Orthop. Unf..

[10] Sanders T.L., Pareek A., Johnson N.R., Stuart M.J., Dahm D.L., Krych A.J. (2016). Patellofemoral Arthritis After Lateral Patellar Dislocation: A Matched Population-Based Analysis. Am. J. Sports Med..

[11] Van Middelkoop M., Bennell K.L., Callaghan M., Collins N.J., Conaghan P.G., Crossley K.M., Eijkenboom J.J., van der Heijden R.A., Hinman R.S., Hunter D.J. (2017). International patellofemoral osteoarthritis consortium: Consensus statement on the diagnosis, burden, outcome measures, prognosis, risk factors and treatment. Semin. Arthritis Rheum..

[12] Duthon V. (2015). Acute traumatic patellar dislocation. Orthop. Traumatol. Surg. Res..

[13] Hasler C.C., Studer D. (2016). Patella instability in children and adolescents. EFORT Open Rev..

[14] Kay R.M., Dennis S., Rethlefsen S., Reynolds R.A.K., Skaggs D.L., Tolo V.T. (2000). The Effect of Preoperative Gait Analysis on Orthopaedic Decision Making. Clin. Orthop. Relat. Res..

[15] Lofterød B., Terjesen T., Skaaret I., Huse A.-B., Jahnsen R. (2007). Preoperative gait analysis has a substantial effect on orthopedic decision making in children with cerebral palsy: Comparison between clinical evaluation and gait analysis in 60 patients. Acta Orthop..

[16] Cook R.E., Schneider I., Hazlewood M.E., Hillman S., Robb J.E. (2003). Gait Analysis Alters Decision-Making in Cerebral Palsy. J. Pediatr. Orthop..

[17] Dewan V., Webb M., Prakash D., Malik A., Gella S., Kipps C. (2019). When does the patella dislocate? A systematic review of biomechanical & kinematic studies. J. Orthop..

[18] Smith T.O., McNamara I., Donell S.T. (2013). The contemporary management of anterior knee pain and patellofemoral instability. Knee.

[19] Barton C.J., Levinger P., Menz H.B., Webster K.E. (2009). Kinematic gait characteristics associated with patellofemoral pain syndrome: A systematic review. Gait Posture.

[20] Arazpour M., Bahramian F., Aboutorabi A., Nourbakhsh S.T., Alidousti A., Aslani H. (2016). The Effect of Patellofemoral Pain Syndrome on Gait Parameters: A Literature Review. Arch. Bone Jt. Surg..

[21] Chesworth B.M., Culham E.G., Tata G.E., Peat M. (1989). Validation of Outcome Measures in Patients with Patellofemoral Syndrome. J. Orthop. Sports Phys. Ther..

[22] Nadeau S., Gravel D., Hébert L.J., Arsenault A., Lepage Y. (1997). Gait study of patients with patellofemoral pain syndrome. Gait Posture.

[23] Paoloni M., Mangone M., Fratocchi G., Murgia M., Saraceni V.M., Santilli V. (2010). Kinematic and kinetic features of normal level walking in patellofemoral pain syndrome: More than a sagittal plane alteration. J. Biomech..

[24] Camathias C., Ammann E., Meier R.L., Rutz E., Vavken P., Studer K. (2020). Recurrent patellar dislocations in adolescents result in decreased knee flexion during the entire gait cycle. Knee Surg. Sports Traumatol. Arthrosc..

[25] Lucas K.C.H., Jacobs C., Lattermann C., Noehren B. (2020). Gait deviations and muscle strength deficits in subjects with patellar instability. Knee.

[26] Schranz C., Belohlavek T., Sperl M., Kraus T., Svehlik M. (2021). Does femoral anteversion and internally rotated gait correlate in subjects with patellofemoral instability?. Clin. Biomech..

[27] Sowiński T., Syczewska M., Kwiatkowski K., Kalinowska M. (2010). Zmiany stereotypu chodu chorych na nawracajace boczne zwichniecie rzepki po osteotomii guzowatości kości piszczelowej zmodyfikowanym sposobem Elmsllie-Trillata. Pol. Merkur. Lek. Organ Pol. Tow. Lek..

[28] Ammann E., Meier R.L., Rutz E., Vavken P., Studer K., Camathias C. (2020). Trochleoplasty improves knee flexion angles and quadriceps function during gait only if performed bilaterally. Knee Surg. Sports Traumatol. Arthrosc..

[29] Clark D.A., Simpson D.L., Eldridge J., Colborne G.R., Information P.E.K.F.C. (2015). Patellar instability and quadriceps avoidance affect walking knee moments. Knee.

[30] Richardson W.S., Wilson M.C., Nishikawa J., Hayward R.S. (1995). The well-built clinical question: A key to evidence-based decisions. ACP J. Club.

[31] Page M.J., McKenzie J.E., Bossuyt P.M., Boutron I., Hoffmann T.C., Mulrow C.D., Shamseer L., Tetzlaff J.M., Akl E.A., Brennan S.E. (2021). The PRISMA 2020 statement: An updated guideline for reporting systematic reviews. BMJ.

[32] Vandenbroucke J.P., von Elm E., Altman D.G., Gøtzsche P.C., Mulrow C.D., Pocock S.J., Poole C., Schlesselman J.J., Egger M., Strobe Initiative (2007). Strengthening the Reporting of Observational Studies in Epidemiology (STROBE): Explanation and Elaboration. PLoS Med..

[33] Von Elm E., Altman D.G., Egger M., Pocock S.J., Gøtzsche P.C., Vandenbroucke J.P. (2008). The Strengthening the Reporting of Observational Studies in Epidemiology (STROBE) statement: Guidelines for reporting observational studies. J. Clin. Epidemiol. JCE Off. J. Int. Clin. Epidemiol. Netw..

[34] Schranz C., Sperl M., Kraus T., Guggenberger B., Kruse A., Habersack A., Svehlik M. (2021). Different Gait Pattern in Adolescence with Patellofemoral Instability.

[35] Berchuck M., Andriacchi T.P., Bach B.R., Reider B. (1990). Gait adaptations by patients who have a deficient anterior cruciate ligament. J. Bone Jt. Surg..

[36] Hart J.M., Ko J.-W.K., Konold T., Pietrosimione B. (2010). Sagittal plane knee joint moments following anterior cruciate ligament injury and reconstruction: A systematic review. Clin. Biomech..

[37] Hurd W.J., Snyder-Mackler L. (2007). Knee instability after acute ACL rupture affects movement patterns during the mid-stance phase of gait. J. Orthop. Res..

[38] Knoll Z., Kiss R.M. (2003). Gait patterns before and after anterior cruciate ligament reconstruction. Knee Surg. Sports Traumatol. Arthrosc..

[39] Rudolph K.S., E Eastlack M., Axe M.J., Snyder-Mackler L. (1998). 1998 Basmajian Student Award Paper: Movement patterns after anterior cruciate ligament injury: A comparison of patients who compensate well for the injury and those who require operative stabilization. J. Electromyogr. Kinesiol..

[40] Werner S. (2014). Anterior knee pain: An update of physical therapy. Knee Surg. Sports Traumatol. Arthrosc..

[41] Wexler G., Hurwitz D.E., Bush-Joseph C.A., Andriacchi T.P., Bach B.R. (1998). Functional Gait Adaptations in Patients with Anterior Cruciate Ligament Deficiency Over Time. Clin. Orthop. Relat. Res..

[42] Christensen T.C., Sanders T.L., Pareek A., Mohan R., Dahm D.L., Krych A.J. (2017). Risk Factors and Time to Recurrent Ipsilateral and Contralateral Patellar Dislocations. Am. J. Sports Med..

[43] Jibri Z., Jamieson P., Rakhra K.S., Sampaio M.L., Dervin G. (2019). Patellar maltracking: An update on the diagnosis and treatment strategies. Insights Imaging.

[44] Fukuchi C., Fukuchi R.K., Duarte M. (2019). Effects of walking speed on gait biomechanics in healthy participants: A systematic review and meta-analysis. Syst. Rev..

